# Eosinophils: old cells, new directions

**DOI:** 10.3389/fmed.2024.1470381

**Published:** 2025-01-16

**Authors:** Alejandra Sanchez Santos, Iovanna Socorro Avila, Helena Galvan Fernandez, Sara Cazorla Rivero, Angelina Lemes Castellano, Carlos Cabrera Lopez

**Affiliations:** ^1^Hospital Universitario de Gran Canaria Dr. Negrín, Respiratory Service, Las Palmas de Gran Canaria, Spain; ^2^Hospital Universitario de Gran Canaria Dr. Negrín, Research Unit, Las Palmas de Gran Canaria, Spain; ^3^Universidad de La Laguna, Research Unit, Santa Cruz de Tenerife, Spain; ^4^Hospital Universitario de Gran Canaria Dr. Negrín, Hematology Service, Las Palmas de Gran Canaria, Spain

**Keywords:** asthma, COPD, eosinophils, subtypes, inflammation

## Abstract

Eosinophils are polymorphonuclear cells that have progressively gained attention due to their involvement in multiple diseases and, more recently, in various homeostatic processes. Their well-known roles range from asthma and parasitic infections to less prevalent diseases such as eosinophilic granulomatosis with polyangiitis, eosinophilic esophagitis, and hypereosinophilic syndrome. In recent years, various biological therapies targeting these cells have been developed, altering the course of eosinophilic pathologies. Recent research has demonstrated differences in eosinophil subtypes and their functions. The presence of distinct classes of eosinophils has led to the theory of resident eosinophils (rEos) and inflammatory eosinophils (iEos). Subtype differences are determined by the pattern of protein expression on the cell membrane and the localization of eosinophils. Most of this research has been conducted in murine models, but several studies confirm these findings in peripheral blood and tissue. The objective of this review is to provide a comprehensive analysis of eosinophils, by recent findings that divide this cell line into two distinct populations with different functions and purposes.

## Introduction

Jones (1) and Brewer (2) first described granular cells, but without appropriate staining techniques, they were unable to correctly characterize or evaluate the various types of granulocytes. Paul Ehrlich, in 1846, using his blood staining technique with coal tar dyes (eosin), successfully described eosinophils based on their strong affinity for this marker (3). In addition to describing the staining properties of their granules, Ehrlich studied their distribution in various species and tissues, concluding that they likely developed in the bone marrow. The discovery of the eosinophil precursor cell took much longer. The higher density of these cells in the bone marrow was demonstrated in 1960 by Rytomaa, but it was not until 1984 that Fischkoff et al. (4) showed that eosinophils and neutrophils share the same precursor: the promyelocytic cell line HL-60. In 1998, it was discovered that the gene EOS47, specifically expressed in bone marrow eosinophils, has a promoter region with binding sites for the transcription factors Myb-Ets, c/EBP, and GATA (5), which are responsible for lineage commitment. Subsequent studies have shown that eliminating the high-affinity GATA-1 binding site in the GATA-1 gene promoter results in the loss of the eosinophil lineage (6).

Another significant finding relates to eosinopoiesis and its regulation. Boyer et al. (7) and Basten and Beeson (8) demonstrated that immunocompetent lymphocytes are responsible for the increased number of eosinophils in peripheral blood during parasitic infections. A few years later, interleukin 5 (IL-5) was isolated as the main protein associated with terminal differentiation, eosinophil production in the bone marrow, their growth, activation, and inhibition of apoptosis (9).

## Granules and degranulation

Eosinophils contain numerous cytoplasmic granules that include specific eosinophilic proteins, cytokines, chemokines, enzymes, and lipid mediators contributing to their function (10). These granules contain four specific proteins stored in secondary granules (major basic proteins, MBP1 and MBP2; eosinophil peroxidase, EPO, eosinophil cationic protein, ECP; and eosinophil-derived neurotoxin, EDN), which can induce tissue damage and dysfunction (11). ECP and EDN are ribonucleases with antiviral activity, with ECP creating voltage-insensitive toxic ion pores in target cell membranes, potentially facilitating the entry of other cytotoxic molecules (12–15). ECP also has additional non-cytotoxic activities, including suppressing T cell proliferative responses, inhibiting immunoglobulin synthesis by B cells, inducing mast cell degranulation, and stimulating airway mucus secretion and glycosaminoglycan production by human fibroblasts (16). MBP directly alters smooth muscle contraction responses by dysregulating M2 and M3 vagal muscarinic receptor function and inducing mast cell and basophil degranulation (17–19). EPO, comprising approximately 25% of the total specific granule protein mass, catalyzes the oxidation of pseudohalides and nitric oxide to form highly reactive oxygen species (hypohalous acids) and reactive nitrogen metabolites (peroxynitrite), which oxidize nucleophilic targets in proteins, promoting oxidative stress and subsequent cell death via apoptosis and necrosis (20–22).

Eosinophils degranulate through four mechanisms: classical exocytosis, compound exocytosis, piecemeal degranulation (regulated), and cytolysis (necrosis). Classical exocytosis refers to the process by which secretory granules release their complete contents into the extracellular space following the fusion of the granule membrane with the plasma membrane. This process encompasses compound exocytosis, which additionally involves the fusion of intracellular granules prior to the subsequent release of their contents into the extracellular environment (23). Piecemeal degranulation (PMD) is a process characterized by the secretion of substances from intracellular granules, facilitated by the transport of vesicles (23). This mechanism allows for the gradual release of granule contents, enabling precise regulation of cellular functions and responses. Cytolysis, the release of granule contents due to cell rupture, involves chromatolysis (disintegration of nuclear chromatin) followed by the rupture of the cell’s plasma membrane. This process leads to the release of membrane-bound eosinophilic granules (FEGs) (24) and is often associated with the formation of eosinophil extracellular traps (EETs) (25).

EETs consist of DNA fibers embedded with granule proteins, such as MBP and ECP (25), or associated with FEGs (23) and eosinophil sombrero vesicles EoSVs (24). The release of EETs has been observed from both live eosinophils and those undergoing cell lysis (EETosis) (26). In recent years, EETosis has gained more attention (25, 27), it drives the release of EETs in tissues and the secretion during several inflammatory diseases (26), playing a critical role in the pathophysiology of severe asthma (28). External stimuli have been suggested to influence EET release, and the extent of release appears to be time-dependent based on exposure duration (25). However, the molecular mechanisms underlying this process remain poorly understood (25). The process of EET formation is associated with the development of Charcot-Leyden crystals (CLCs), which are composed of the protein galectin-10. These crystals serve as a biomarker of eosinophil involvement in conditions such as asthma, allergic rhinitis, and other forms of eosinophilic inflammation (26, 29).

In areas of eosinophilic inflammation characterized by the presence of FEGs and occasionally CLCs, EoSVs are often observed near or intermingled with extracellular, expanded, and highly decondensed chromatin (24). This represents an ultrastructural hallmark of the late stage of EETosis (26). EoSVs are thought to be crucial intermediaries in this process. The total number of EoSVs increases when eosinophils are exposed to inflammatory stimuli in activated eosinophils both *in vitro* and *in vivo* (24). In tissues affected by eosinophilic cytolytic inflammation, extracellular EoSVs are present; however, their clinical significance in eosinophil-associated diseases remains unclear (24).

Different cytokines have distinct effects on eosinophil degranulation, influencing both the type and extent of granule release (27, 30, 31). The nature and extent of eosinophil degranulation can vary depending on the specific cytokine stimulation the cell receives (32, 33). For example, TNF-α is a potent pro-inflammatory cytokine that induces oxidative stress and membrane destabilization in eosinophils, promoting cytolysis (23). However, it is hypothesized that each degranulation form corresponds to the specific function the eosinophil is performing. For instance, during PMD, eosinophils selectively release components of their specific granules (34). IFN-γ is associated with Th1 responses and can modulate eosinophil degranulation in a more controlled manner. It often acts as a suppressor of eosinophil degranulation, particularly in allergic inflammation (32, 35). However, human eosinophil activation by IFN-γ promotes the mobilization of RANTES (CCL5) derived from granules to the cell periphery without releasing cationic proteins (36, 37). Regulated exocytosis occurs through the formation of a docking complex composed of soluble N-ethylmaleimide-sensitive factor attachment protein receptors (SNAP receptors or SNARE) located on the vesicle (v-SNARE) and the target membrane (t-SNARE) (38).

## Migration

Under normal conditions, eosinophils migrate from the bone marrow to specific organs, primarily the gastrointestinal system. Most eosinophils reside in the non-esophageal portion of the intestine. Other target organs include the uterus and mammary glands of young women, the thymus, adipose tissue, and the lungs.

Traditionally, eosinophils have been associated with the inflammatory response to helminth infections and allergic diseases. However, it is now recognized that they have more varied functions depending on the tissue in which they are found. Studies in mice have shown that, under stable conditions, eosinophils play a homeostatic role in these tissues. In the intestine, they are involved in the IgA response and mucus production (39); in the mammary glands, they seem to play a role in development (40), while in adipose tissue, they are associated with insulin sensitivity and the transition to brown fat (39).

**FIGURE 1:**
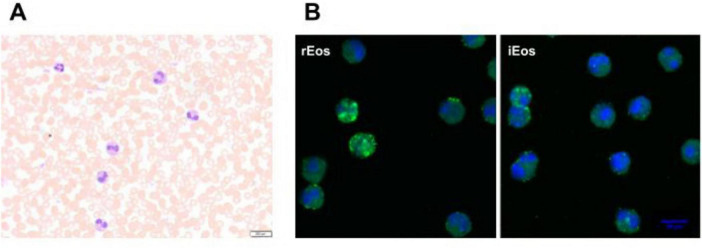
Human eosinophils in peripheral blood from an asthma patient. **(A)** Blood eosinophils were directly stained with Hematoxylin-eosin before sorting (40× magnification). **(B)** Representative confocal microscopy photographs of blood rEos and iEos after FACS sorting, following the gating strategy of Cabrera López et al. (93). Eosinophils were stained for CD62L (green) and DAPI (blue, nucleus). DAPI, 4′,6-diamidino-2-phenylindole; 63× magnification. rEos: resident eosinophils, iEos: inflammatory eosinophils.

Eosinophils are tissue cells, therefore typically constitute less than 5% of the total leukocytes in the blood (39) (Figure 1A). *In vivo* studies have shown that the residence time of eosinophils in the bloodstream is quite short, approximately 8–10 h, although the range can vary from 3 to 24 h (41–43). In contrast, their persistence in tissues is longer, with a half-life of 36 h in the lung and up to 6 days in the intestine, thymus, and uterus (41). The tissue longevity of eosinophils appears to be related to the expression of CD11c, which is expressed by eosinophils in the thymus, uterus, and intestine but not by those in the blood and lung. This longevity also depends on the inhibition of apoptosis mediated by IL-5 (41, 44, 45).

## Cytokines and chemokines

The recruitment of eosinophils into tissues is driven by the eotaxin family, primarily eotaxin-1 (CCL11), a chemokine produced mainly by epithelial cells, endothelial cells, fibroblasts, and monocytes in response to inflammatory signals such as IL-4, IL-13, and TNF-α (46–49). The differential chemotactic potential and expression profiles of CCL11, eotaxin-2 (CCL24), and eotaxin-3 (CCL26) suggest that they play distinct roles over time. CCL11 is the most potent eosinophil chemoattractant of the three (50). CCL11 may act early in an inflammatory response to recruit eosinophils quickly, while CCL24 and CCL26 might sustain eosinophil accumulation and inflammation over longer periods. Its strong affinity for CCR3 and the efficient signaling it induces lead to rapid and robust eosinophil migration (51). The loss of CCR3, the principal receptor for eotaxin-1 (52, 53), results in defective localization of eosinophils in tissues, particularly in the intestine, but does not affect the cell count in the lung or thymus (54), suggesting that eotaxin-1 may act through alternative receptors such as CCR5 (55). Interleukins, IL-5 and IL-13, released by T lymphocytes and type 2 innate lymphoid cells (ILC2) (9, 56–59), can also promote eosinophil trafficking under normal conditions (60, 61), albeit to a lesser extent than eotaxin-1. IL-13 enhances the production of eotaxin-3 (56), while IL-5 promotes eosinophil generation from bone marrow progenitors, increases their sensitivity to eotaxin-1, and maintains their survival (9, 57, 58).

IL-5, the cytokine most specific to the eosinophil lineage, is essential for eosinophil production in steady-state conditions. The recruitment of resident eosinophils to tissues is independent in the lungs, partially dependent in the gastrointestinal tract and uterus, and completely dependent in adipose tissue on local IL-5 production (59–63).

In allergic and reactive diseases, eosinophils have been identified as significant sources of IL-5, IL-13, IL-25, and CCL26, contributing to the Th2-skewed immune response and subsequent eosinophilic inflammation (64). Among the eotaxins, CCL26 displays the weakest chemotactic activity, despite also binding to CCR3 with lower affinity and eliciting a reduced chemotactic response. CCL26 is upregulated by IL-13 and is predominantly expressed in airway epithelial cells during allergic inflammation. Although its role in eosinophil recruitment is more limited compared to CCL11 and CCL24, it is thought to play a role in asthma (65). In the lung, IL-4 and IL-13 secreted locally are responsible for increasing endothelial adhesiveness by upregulating VCAM-1 (66) and inducing CCL11 secretion by bronchial epithelial cells (50), which promotes greater eosinophil recruitment into the tissue.

The enhanced tissue survival of eosinophils is mediated by IL-5, IL-3 and granulocyte-macrophage colony-stimulating factor (GM-CSF). These cytokines are essential hematopoietic signals that regulate eosinophil development and differentiation within the bone marrow (10, 67). IL-3 is primarily involved in the early expansion of eosinophil progenitor cells, while IL-5 is crucial for the terminal differentiation of these cells (56). GM-CSF further supports the maturation and survival of both progenitors and mature eosinophils. IL-3 signals through the IL-3 receptor (IL-3R), composed of a specific α-subunit (IL-3Rα) and a shared β-common chain (βc), the latter of which is also utilized by GM-CSF and IL-5 (67). Upon ligand binding, IL-3R activates several intracellular pathways, including JAK/STAT, MAPK, and PI3K, which act in synergy with IL-5 and GM-CSF (68). Dysregulation of IL-3 and GM-CSF signaling pathways is implicated in eosinophilic disorders, contributing to excessive eosinophil survival, tissue damage, and chronic inflammation (67, 69).

Eosinophils promote humoral immunity by priming B cells (39) and play a central role in type 2 immunity, including antigen presentation to CD4+ T cells and secretion of granular contents containing type 2 mediators, such as IL-4, IL-5, and IL-13 (39), thereby closely regulating Th1 and Th2 immunity (70).

## Eosinophil activation

Eosinophils are terminal effector cells that degranulate and release highly cytotoxic substances when activated. In the case of infection, these granular proteins act directly against parasites; however, in allergic situations, they contribute to tissue destruction, as seen in patients with atopic asthma, where the number of eosinophils in the bronchi correlates with lung epithelial damage (71, 72). As mentioned above, fully activated eosinophils can also expel EET composed of mitochondrial DNA and granular proteins, which are destructive to tissues (73). In this way, eosinophils, like neutrophils, can trap and kill other types of microorganisms.

However, this cytotoxic reaction occurs only under inflammatory conditions when eosinophils are highly stimulated by cytokines such as interferon gamma (IFN-γ) and require high levels of IL-5 to induce the formation of a DNA net (73). EET formation has been observed to be triggered by eosinophil activation through IL-5 and thymic stromal lymphopoietin (TSLP) (74). Future studies are needed to better understand how the molecular mechanisms of EET production are regulated (28).

Besides these inflammatory functions, eosinophils also play a beneficial role in regulating and modulating immune responses, partly by synthesizing and secreting a wide array of cytokines and immune mediators (75). They do this, at least in part, by synthesizing and secreting a surprisingly broad spectrum of different cytokines and immune mediators (10).

The actions of eosinophils go beyond the secretion of toxic proteins. Eosinophil activation promotes the secretion of various pro-inflammatory cytokines (IL-2, IL-4, IL-5, IL-10, IL-12, IL-13, IL-16, IL-18, and TGF-α/β), chemokines (RANTES and eotaxin-1), and lipid mediators (platelet-activating factor and leukotriene C4, LTC4) (76). These molecules have pro-inflammatory effects, positively regulating adhesion systems, modulating cell trafficking, activating and regulating vascular permeability, mucus secretion, and smooth muscle constriction. Eosinophils can initiate antigen-specific immune responses by acting as antigen-presenting cells (APC) to major histocompatibility complex class II and co-stimulatory molecules (CD40, CD80, CD86).

Eosinophils are also activated by epithelial-derived innate cytokines (TSLP and IL-33), promoting their recruitment by amplifying Th2 responses and stimulating ILC2 cells to secrete IL-5, IL-4, and IL-13, as well as by stimulating T lymphocytes. In addition to promoting Th2 responses, TSLP and IL-33 act directly on eosinophils, preventing apoptosis through direct activation of the TSLP receptor (TSLPR) present on eosinophils (77, 78).

## Eosinophil subtypes

In recent years, several publications have classified eosinophils into different subtypes. It remains unclear whether these represent the same cell line at different activation stages or, as occurs with Th1 or Th2 lymphocytes, distinct cells with different properties secreted from the bone marrow. A pivotal study by Mesnil et al. (79) using an asthmatic murine model delineates the distinction between resident, homeostatic or physiological eosinophils (rEos) and inflammatory eosinophils (iEos). This research conducted multiple experiments in the lungs and blood of mice, revealing clear differences between populations in different models (allergic asthmatic mice vs. healthy mice). In mice, this differentiation is characterized by nuclear shape, membrane proteins, and cell localization. rEos exhibit most typical eosinophil features, including red-staining granules containing specific proteins (e.g., MBP, EPO) and combined expression of CCR3, Siglec-F, and CD125 (the IL-5 receptor α subunit) (39, 45, 80). They can also express CD11b (intestine, thymus, and adipose tissue), F4/80 (mammary glands, lung, and adipose tissue), CD69 and CD44 (intestine and thymus) (45, 79–85). Most tissue rEos have a segmented nucleus and express CD11c (12, 75, 82–86).

Lung mice rEos are an exception and resemble resting blood eosinophils with a ring-shaped nucleus, express CD62L, show intermediate Siglec-F levels, and are CD11c negative (6, 79, 82, 85–87). In mice, such characteristics, especially the ring-shaped nucleus, indicate cellular immaturity (88, 89), suggesting that lung rEos retain an immature phenotype upon dissemination to the lungs. These eosinophils undergo gradual degranulation and are capable of phagocytosis, demonstrating their functionality.

**FIGURE 2:**
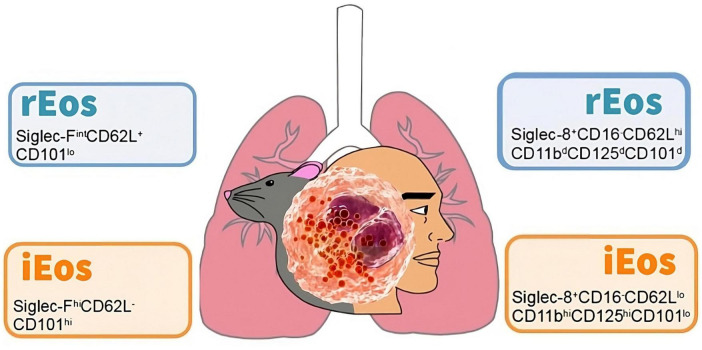
Cell phenotyping of blood eosinophils subtypes in mouse and human. Data taken from the studies of Mesnil et al. (79) (mouse) and Cabrera López et al. (93) (human). rEos: resident eosinophils, iEos: inflammatory eosinophils.

Interestingly, the number, localization, and morphological, phenotypic, and transcriptomic characteristics of lung rEos remain unchanged and differ from iEos during allergic airway inflammation. iEos, abundantly recruited to the lungs during allergen exposure episodes, are defined as SiglecF*^hi^*CD62L^–^CD101*^hi^* cells with a segmented nucleus (CD101 is an iEos marker not expressed in lung rEos). These observations support the theory of rEos and iEos, suggesting that similar subsets exist in the blood of asthmatic mice, indicating differentiation occurs even before tissue recruitment. This study also conducted a human experiment comparing lung tissue from healthy individuals with sputum from asthmatic patients (79). The results showed that parenchymal rEos in non-asthmatic human lungs (Siglec-8^+^CD62L*^hi^*IL-3R*^lo^* cells) are phenotypically distinct from iEos isolated from asthmatic patient sputum (Siglec-8^+^CD62L*^lo^*IL-3R*^hi^* cells), confirming mouse findings in humans (Figure 2).

Other studies have validated Mesnil et al. (79) proposed pattern in horses (90) and humans (91–95) (Table 1). Matucci et al. (91) focused on different eosinophil subpopulations in peripheral blood and nasal polyps in patients with severe eosinophilic asthma (SEA) with chronic rhinosinusitis with nasal polyps (CRSwNP). They recruited 23 SEA patients (14 with CRSwNP), comparing them with 15 non-severe asthma patients (NSEA), 15 allergic rhinitis without asthma patients, and 15 healthy volunteers. They also studied eotaxin-3 and eotaxin-1 expression in nasal polyps. They observed an increase in peripheral blood eosinophils in SEA patients (Siglec8^+^CD45^+^CD16^–^), revealing two eosinophil subtypes based on CD62L expression across all groups. There was a higher number of CD62L*^lo^* eosinophils in SEA patients compared to controls, expressing high CCR3, CD69, and low CD125 (IL-5R), CRTH2, CD86, CD28, CD101, and VLA-4 levels. Nasal polyps had a higher proportion of CD62L*^lo^* eosinophils than peripheral blood. Surface expression of IL-3R, IL-5R, CD69, and CD86 was significantly higher in CD62L*^lo^* eosinophils from nasal polyps compared to blood. Further, eotaxin-3 expression correlated with the percentage of CD62L*^lo^* eosinophils in nasal polyps. In relation to what was previously published, CD62L*^lo^* was associated with iEos and CD62L*^bright^* with rEos (79, 96).

**TABLE 1:** Different antibodies used in cell phenotyping eosinophil panels.

Mesnil et al. (79)	Januskevicius et al. (96)	Matucci et al. (91) and Vultaggio et al. (92)	Cabrera López et al. (93)	Mycroft et al. (94)	Fricker M et al. (95)
CD45		CD45	CD45	CD45	CD45
Siglec-8		Siglec-8	Siglec-8	Siglec-8	Siglec-8
CD62L	CD62L	CD62L	CD62L	CD62L	CD62L
CD101	CD101	CD101	CD101		
CD123		CD123	CD123		
CD125		CD125	CD125	CD125	CD125
		CD16	CD16	CD16	
			CD11b	CD11b	CD11b
		CD193		CD193	
		CD86, CD28, CD69, CD294		CD66b, CD14	

The Vultaggio et al. (92), study is notable for correlating iEos presence with clinical outcomes, is undoubtedly one of the most interesting published so far. It examined the relationship between iEos (characterized by CD62L*^lo^*) in blood and the severity of severe eosinophilic asthma, evaluating the impact of mepolizumab on iEos (92). They recruited 112 patients: 51 naive and 61 previously treated with biologics. They analyzed 19 naive patients before and after 100 mg SC mepolizumab/4 weeks treatment, and 23 patients already on mepolizumab at study start. *In vitro* effects of IL-5 and mepolizumab on CD62L expression were also evaluated. There was a significant correlation between CD62L*^lo^* cells and better ACT scores in asthma, lower SNOT-22 scores in nasal polyposis (better asthma and nasal polyposis control in patients with low CD62L*^lo^* eosinophils), as well as exacerbations in untreated patients. The Naive group showed a reduction in CD62L*^lo^* with an increase in CD62*^bright^* proportion after mepolizumab treatment, associating with improved asthma control, resembling healthy volunteer rEos/iEos proportions. *In vitro*, IL-5 and anti-IL-5 regulated CD62L expression on eosinophils. IL-3, GM-CSF, IL-33, TSLP, and TNF-α modulated CD62L expression, but not IL-4.

Fricker et al. (95) analyzed eosinophil subpopulations in patients with severe asthma, finding results similar to those of Matucci et al. (91) and Cabrera López et al. (93). Additionally, a longitudinal analysis was conducted in patients undergoing treatment (*n* = 30) at two timepoints (4–24 weeks) post-initiation of mepolizumab (*n* = 20) or benralizumab (*n* = 10). Similar to Vultaggio’s findings, both mepolizumab and benralizumab effectively iEos to a comparable extent. Mepolizumab, however, specifically depleted iEos while preserving a residual population of rEos in patients with severe asthma, whereas benralizumab depleted both subtypes. This confirms that an increase in the proportion of circulating iEos is associated with poorer asthma control (95).

The Cabrera López et al. (93), study highlights the presence of iEos in asthmatic patients (over 20% of the total count) with minimal percentages (less than 1%) in healthy subjects, smokers without chronic obstructive pulmonary disease (COPD), and COPD subjects. In this study, it was observed that iEos are independent of disease severity, treatment, and exacerbations in patients with COPD. Additionally, the proportion of iEos in asthmatic subjects is independent of the total blood eosinophil count. For instance, patients with only 250 eosinophils per microliter can have up to 45% iEos. This finding may explain the discrepancy between the number of eosinophils in the blood and in the tissues.

Cabrera López et al. (93) analyzed freshly unfractionated blood (100 μl) from 10 stable subjects of four groups: (COPD), asthma, smokers without COPD, and healthy volunteers; data were validated in 59 patients with COPD and in 17 patients with asthma. Cell phenotyping was according to the Mesnil criteria and other crucial proteins as CD125 and CD11b (Table 2). iEos were identified following the algorithm: CD45^+^Siglec8^+^CD16^–^CD62L*^lo^*CD11b*^hi^*CD125*^hi^*CD101*^lo^* and rEos were identified as CD45^+^Siglec8^+^CD16^–^CD62L*^hi^*CD11b*^d^*CD125*^d^* CD101*^d^* by flow cytometry and confocal microscopy (Figure 1B).

**TABLE 2:** Expression and function of the crucial surface markers of the eosinophils and their importance in flow cytometry.

Surface markers	Expression	Function	Eosinophil Flow cytometry
CD45 Phosphotyrosine phosphatase	Expressed on nucleated hematopoietic human cells, lymphocytes, monocytes, eosinophils, basophils, and neutrophils, except erythrocytes and platelets.	-CD45 is a transmembrane glycoprotein with tyrosine phosphatase activity. Can be expressed as one of at least five isoforms (180 to 220 kDa) by alternative splicing of exons comprising the extracellular domain, depending on cell type and activation state. -Functions as a tyrosine phosphatase, regulating T and B cell receptor signaling. Critical for the development, differentiation, and activation of immune cells.	CD45 is a pan-leukocyte marker, essential in eosinophils (98).
CD11b (Integrin subunit αM)	Highly expressed on monocytes, macrophages, granulocytes, dendritic cells, and NK cells. Also found on some T cells during activation.	-CD11b, in combination with CD18, forms a αMβ2 heterodimer (Mac-1) (99). -Plays a crucial role in cell adhesion, phagocytosis, migration, and inflammation. Involved in innate immune responses. Important in inflammation, tissue injury, and autoimmunity. -Upregulated during infections and inflammatory conditions. Upregulated in eosinophil activation during inflammatory responses (93)	Used as a general granulocytes marker, it is one of the most critical markers to differentiate eosinophil subtypes (93)
CD16 (FcγRIII)	Found on NK cells, macrophages, neutrophils, and some T cells. No expression on eosinophils.	Functions as a low-affinity receptor for the Fc portion of IgG antibodies, mediating antibody-dependent cellular cytotoxicity (ADCC) (98). - Plays a role in phagocytosis of immune complexes. Central to NK cell function and phagocytes -The absence of this receptor in eosinophils may be due to the low phagocytic capacity compared to the neutrophil.	Eosinophils are differentiated from neutrophils by lack of CD16 expression (98)
Siglec-8 (Sialic acid-binding immunoglobulin-like lectin-8)	Primarily expressed on eosinophils, mast cells, and basophils (100).	-Siglec-8 are involved in eosinophil survival and apoptosis when bound to sialylated glycan ligands (100, 101), positioning it as a therapeutic target for eosinophil-associated conditions such as asthma and eosinophilic esophagitis	Highly expressed on eosinophils. Used as a eosinophils marker (100)
CD62L (L-selectin)	Found on leukocytes, including naive and memory T cells, B cells, monocytes, and granulocytes.	-CD62L is an adhesion molecule that facilitates leukocyte transmigration. -Mediates the capture and rolling of leukocytes on the endothelium at sites of inflammation, facilitating their migration to secondary lymphoid organs and inflamed tissues (102), playing a critical role in both adaptive immune responses and inflammation. -Data suggest that shedding of L-selectin from the surface of granulocytes occurs in the peripheral circulation as a prelude to diapedesis of granulocytes into peripheral tissues (103). Downregulated upon activation during allergic responses and chronic inflammation.	Expressed on circulating eosinophils (103), it is one of the most critical markers to differentiate eosinophil subtypes (79).
CD125 (IL-5Rα)	Expressed predominantly on eosinophils, basophils, and certain activated T cells.	-Interleukin 5 receptor (IL-5R) complex comprises an IL-5-binding protein, commonly referred to as IL-5Rα (CD125), and a ß-chain (CD131) (56). -CD125 is crucial for eosinophil proliferation, immune responses activation, and survival in response to IL-5 (104). - Involved in allergic immune responses and eosinophilic disorders, such as asthma eosinophilic esophagitis, hypereosinophilic syndrome and allergic rhinitis, making it a target in the treatment of eosinophilia-associated disorders.	Highly expressed on eosinophils, is one of the most critical markers to differentiate eosinophil subtypes (93).
CD101	Expressed on dendritic cells, monocytes, macrophages, granulocytes, memory T cells, and regulatory T cells (Tregs). (13).	-Modulates immune activation by regulating T cell proliferation and Treg function. Its expression has been linked to modulating the severity of autoimmune diseases and inflammatory responses. Contributes to the maintenance of immune tolerance and immune suppression. -The expression of CD101 on myeloid cells induces the release of IL-10 and TGF-β, but has no effect on the release of inflammatory cytokines. A (relative) lack of CD101 signals promotes the expansion of T cells and the induction of an inflammatory cytokine profile (105).	Low expression in eosinophils. However is one of the most critical markers to differentiate eosinophil subtypes (93)
CD123 (IL-3Rα)	Expressed on plasmacytoid dendritic cells (pDCs), basophils, monocytes, and some hematopoietic progenitor cells. Expressed at low levels in eosinophils	-Cytokine receptor important for the proliferation and differentiation of hematopoietic cells, potentially influencing activation, survival and differentiation of eosinophils via IL-3-mediated signaling. Expressed during all stages of eosinophil maturation, but the highest levels are found in mature eosinophils (67). - Regulates the activation of plasmacytoid dendritic cells, which are involved in producing large amounts of type I interferons in response to viral infections.	While not a major marker in eosinophils, it may play a role to differentiate eosinophil subtypes (79)

For the purposes of this review, these asthmatic patients were divided into SEA and NSEA. The observed variations in the proportions of human blood eosinophils subpopulations may be attributed to divergent processing methods of the samples in the different studies. Specifically, Januskevicius et al. (96) demonstrated higher levels of iEos in SEA patients (Table 3), likely due to the magnetic selection of eosinophils using CD62L as a marker. This selection process may lead to the downregulation of the protein following interaction with the magnetic beads.

**TABLE 3:** Percentages of rEos and iEos in human peripheral blood.

	Eosinophils count, cell/μL	% iEos	Eosinophils count, cell/μL	% iEos	Eosinophils count, cell/μL	% iEos
NSEA	530 ± 80	37.2 ± 5.8	205 ± 3	3.28 ± 1.8	380 ± 164	21.5 ± 16.6
SEA	680 ± 110	36.2 ± 3.8	604 ± 79	12.3 ± 3.5	730 ± 91	30.1 ± 13.1
Healthy	170 ± 20	48.7 ± 5.9	109 ± 22	2.25 ± 1.5	203 ± 92	0.67 ± 1.72
COPD					239 ± 181	0.7 ± 1.1
	Januskevicius et al. (96)		Matucci et al. (91) and Vultaggio et al. (92)		Cabrera López et al. (93)	

NSEA, non-severe asthma patients; SEA, severe eosinophilic asthma; COPD, chronic obstructive pulmonary disease.

Conversely, the proportions reported by Matucci et al. (91), Vultaggio et al. (92), and Cabrera López et al. (93) exhibit greater similarity. However, discrepancies persist that could be linked to different methods employed. Matucci et al. (91) and Vultaggio et al. (92) observed a lower percentage of iEos in their studies compared to Cabrera López et al. (93) (Tables 3, 4); a difference that cannot be explained by the analysis algorithm alone. It is possible that there could be a loss of cellularity due to the methodology employed: Ficoll (91, 92) vs. lysis (93), under the hypothesis that the latter method subjects eosinophils to less stress than the Ficoll method, resulting in less loss of eos especially iEos. Furthermore, it is known that in the Canary Islands there is an increase in the number of eosinophils in the blood due to their weather conditions, which could explain the differences found between iEos in SEA and NSEA with other studies.

**TABLE 4:** Differences expression of iEos markers in human blood, nasal polyp and lung.

iEos	Blood	Nasal polyp	Lung
CD62L	Low	Low	Low
CD125	High	High	
CD11b	High		
CD101	Low	High	
CD123	Low	High	High

Data taken from the studies of Cabrera López et al. (93), Matucci et al. (91), and Mesnil et al. (79), respectively.

## Possible implications in asthma and COPD of the different eosinophil’s subpopulations

The concept of iEos and rEos is novel. There is limited evidence regarding the mechanisms and roles these cells play in various diseases. No studies have been conducted on these cellular phenotypes in exacerbations or in patients treated with monoclonal antibodies other than mepolizumab and benralizumab. Furthermore, their functional roles have not been published, and their potential contributions remain speculative. This has been highlighted by the EAACI task force paper on new molecular insights and clinical functions of eosinophils states (97), which calls for research in this topic for the next years. However, several thoughts arise when addressing the possible role of the eosinophil’s subpopulations. One possibility is that identifying a threshold of iEos (probably a 8–10% would be adequate) may be sufficient to categorize an asthmatic patient as having a Th2-high endotype. It is well established that approximately 20% of patients with severe asthma exhibit discordance between blood and tissue eosinophils. This discrepancy might be explained by different eosinophil subpopulations. As exposed previously, asthmatic patients can have a low blood eosinophil count but a high proportion of them can be iEos. This could be the case of the iEos found in non-eosinophilic asthma patients in the study of Fricker et al. (95). We speculate that iEos, due to their molecular surface markers, are the ones driven to the inflammation site. iEos in the blood may serve as a surrogate marker of a Th2 signal, even when the total blood eosinophil count does not exceed 250 cells/mm^3^. This could allow iEos to endotype severe asthma patients, pointing out candidates for biological therapy with anti-IL-5/IL-5R agents even though they do not have a high eosinophil count in peripheral blood. This might also explain why Tezepelumab is effective in non-Th2 asthma. Asthma has traditionally been classified as Th2-high based on blood eosinophil counts rather than tissue eosinophils. There could be a subset of patients with low blood eosinophil counts but elevated eosinophil levels in the bronchi, who may respond well to treatments like Tezepelumab. However, this could represent only part of the explanation, as Tezepelumab affects multiple cell types and mechanisms beyond IL-5 inhibition. According to data from Vultaggio et al. (92) these eosinophil subpopulations may be more predictive of symptom control (asthma and nasal polyposis) than the total blood eosinophil count and could maybe serve as a biomarker of control in patients treated with mepolizumab.

Studies of biological therapies have been disappointing in COPD. Only dupilumab can reduce exacerbations so far and the population where it works better is in those who have high eosinophil blood count, high FENO and IgE. It is necessary to define accurately the COPD patient suitable for monoclonal antibodies in order to achieve a good therapeutic response. Eosinophil’s subpopulations may help identify those who are suitable candidates for anti-eosinophilic treatments. In the study by Cabrera López et al. (93) we found that COPD patients, even those with elevated eosinophil blood counts, had less than 1% of iEos. Identifying COPD patients with a significant percentage of iEos could be highly useful for selecting those who are more likely to respond to monoclonal antibody therapies.

Another gap is if the proportion of iEos and rEos could vary between stable states and exacerbations. iEos could potentially increase during an exacerbation both in asthma and COPD. Such findings could also have important implications for identifying candidates for biological therapies.

## Conclusion

Eosinophils are granulocytic cells historically viewed as purely inflammatory and defensive, often associated with pathological conditions. In recent years, this perception has evolved as research has uncovered their homeostatic roles and synergistic interactions with other immune cells. Recent advancements have demonstrated the existence of different eosinophil subtypes and their potential association with disease severity. However, several questions remain unanswered: Are these true subtypes or merely activated cells? Are they generated in this form in the bone marrow, or do they differentiate later? Can they serve as biomarkers for the use of monoclonal antibodies in asthma and COPD? Do they function similarly when stimulated? Is their genetics similar? Most studies on eosinophils have treated them as a homogeneous population without distinguishing subtypes. To address these questions, it is essential to conduct subtype-specific investigations, as previous studies that did not differentiate subtypes are less comparable. Future research should focus on resolving these issues, which could significantly improve the characterization of patients with eosinophilia and facilitate the development of personalized medicine.
